# High-performance green flexible electronics based on biodegradable cellulose nanofibril paper

**DOI:** 10.1038/ncomms8170

**Published:** 2015-05-26

**Authors:** Yei Hwan Jung, Tzu-Hsuan Chang, Huilong Zhang, Chunhua Yao, Qifeng Zheng, Vina W. Yang, Hongyi Mi, Munho Kim, Sang June Cho, Dong-Wook Park, Hao Jiang, Juhwan Lee, Yijie Qiu, Weidong Zhou, Zhiyong Cai, Shaoqin Gong, Zhenqiang Ma

**Affiliations:** 1 Department of Electrical and Computer Engineering, University of Wisconsin–Madison, 1415 Engineering Drive, 3445 Engineering Hall, Madison, Wisconsin 53706, USA; 2 Department of Material Sciences and Engineering, University of Wisconsin–Madison, Madison, Wisconsin 53706, USA; 3 Forest Products Laboratory, USDA Forest Service, Madison, Wisconsin 53726, USA; 4 School of Electronic Engineering, University of Electronic Science and Technology of China, Chengdu 611731, China; 5 Department of Electrical Engineering, University of Texas-Arlington, Arlington, Texas 76019, USA; 6 Department of Biomedical Engineering and Wisconsin Institutes for Discovery, University of Wisconsin–Madison, Madison, Wisconsin 53706, USA

## Abstract

Today's consumer electronics, such as cell phones, tablets and other portable electronic devices, are typically made of non-renewable, non-biodegradable, and sometimes potentially toxic (for example, gallium arsenide) materials. These consumer electronics are frequently upgraded or discarded, leading to serious environmental contamination. Thus, electronic systems consisting of renewable and biodegradable materials and minimal amount of potentially toxic materials are desirable. Here we report high-performance flexible microwave and digital electronics that consume the smallest amount of potentially toxic materials on biobased, biodegradable and flexible cellulose nanofibril papers. Furthermore, we demonstrate gallium arsenide microwave devices, the consumer wireless workhorse, in a transferrable thin-film form. Successful fabrication of key electrical components on the flexible cellulose nanofibril paper with comparable performance to their rigid counterparts and clear demonstration of fungal biodegradation of the cellulose-nanofibril-based electronics suggest that it is feasible to fabricate high-performance flexible electronics using ecofriendly materials.

## Abstract (web summary)

The rapid evolution of consumer electronics means that out-of-date devices quickly end up in the scrap heap. Here, the authors fabricate electrical components using biodegradable and flexible cellulose nanofibril paper—a natural sustainable resource derived from wood.

## Graphical abstract

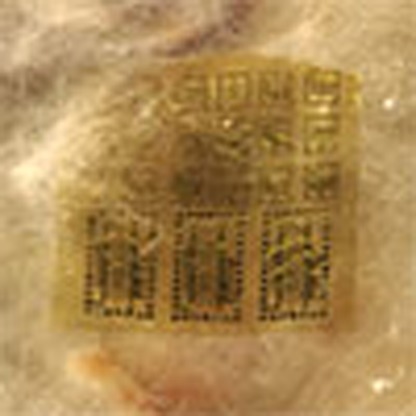

Consumer electronics, particularly portable electronics, such as smart phones, tablets, and so on together with their supported Big Data industry, have helped sustain the economic growth in a majority of the world. However, rapid technological advances have led to a significant decrease in the lifetime of consumer electronics and also rapid consumption of non-renewable natural resources. On average, cell phones are used for <18 months and computers are used for <3 years before being replaced^1^,^2^. In fact, in 2007, it was estimated that over 426,000 cell phones (most of them were still functional) and 112,000 computers were discarded every day in the US, totalling 3.2 million tons of electronic waste generated per year^3^,^4^. These discarded electronics increase the demand for landfill space and may also cause some serious environmental concerns^4^,^5^,^6^,^7^. Thus, the use of biodegradable materials in electronics can reduce the accumulation of persistent solid waste, thereby benefiting our living environment. Moreover, the fabrication of many chips in portable electronic devices involves the consumption of precious materials. In a typical semiconductor electronic chip, the active region comprises the top thin layer and is only a small portion of the chip, whereas the bottom substrate that holds the chip consists of more than 99% of the semiconductor materials^8^. In microwave chips for wireless functions, besides the waste of the bottom substrate, only a tiny fraction of the lateral chip area is used for the required active transistors/diodes with the rest being used only for carrying other non-active components. Some toxic semiconductor materials like gallium arsenide (GaAs)^9^,^10^ are widely used in high speed communication devices, such as cell phones and tablets, and can lead to a significant amount of hazardous materials and high cost in applications that require sparse areal coverage, such as monolithic microwave integrated circuits (MMIC). To minimize the usage of semiconductors, fully formed electronic devices can be fabricated on a sacrificial material in a dense array format, where each micro-scale device can be released and transfer printed onto any type of substrate, including biodegradable flexible substrates. The feasibility of such an approach has been demonstrated in several reports, where devices are fabricated on a wafer that enables deterministic assembly of individual devices on foreign substrates via the transfer printing process^11^,^12^,^13^,^14^. Utilizing this technique, the handling substrate is no longer necessary and the substrate can be re-used for further growth of active layers on the depletion of all devices. This is especially useful in GaAs-based electronics where the chemical extraction of GaAs from discarded waste is prohibitively expensive and dangerous due to the presence of arsenic^9^,^10^. To obtain degradable capabilities, new materials that are non-toxic and biodegradable in nature, such as paper, silk and a number of synthetic polymers, have been explored as electronic substrates^15^,^16^,^17^,^18^,^19^. Many groups have reported electronics on paper by depositing organic semiconductors, but while the concept is very interesting in that it is flexible and degradable, the performance of such electronics falls behind the requirements of state-of-the-art electronics^20^,^21^,^22^. Another promising approach developed by Hwang *et al*.^17^,^19^ was to use biodegradable silk combined with high-performance inorganic semiconductors. Despite their excellent degradation capability, biocompatibility, and performance, silk-based electronics are highly vulnerable to water and solvents. In addition, the suitability of silk for microwave applications is unknown. It should also be noted that only silicon (Si)-based electronics were demonstrated on biodegradable silk substrate. However, Si has a low mobility compared with GaAs, which limits its use in microwave electronics. Cellulose nanofibril (CNF) is an ecofriendly material as it is completely derived from wood^23^,^24^. With its high transparency^25^,^26^ and flexibility^27^, as well as desirable electrical properties^28^, CNF is an ideal candidate as an alternative and ecofriendly substrate for electronics. A number of groups have reported on CNF-based electronics^29^,^30^,^31^,^32^. However, whether the biodegradable CNF substrate exhibits suitable radio frequency (RF) properties for microwave-based consumer electronics applications has been unknown.

In the following, we explore the microwave applications of CNF and demonstrate high-performance electronics that are comparable to existing state-of-the-art electronics, including process-complicated GaAs-based microwave level electronics where the operating frequency is beyond gigahertz, as well as Si-based digital electronics, on CNF substrates. Fungal biodegradation of these CNF-based electronics, for the purpose of cycling degraded CNF back for forestry fertilizer, was also carried out to show the decaying process over time. While transfer printing techniques and CNF paper are used to realize various high-performance flexible electronics in this work, what is described is a new, much more sustainable, green electronic chip concept to address the societal impact of today's economically important yet environmentally unsustainable consumer electronics, based on the important properties that were discovered from CNF.

## Results

### CNF film and its characteristics

**Figure 1:**
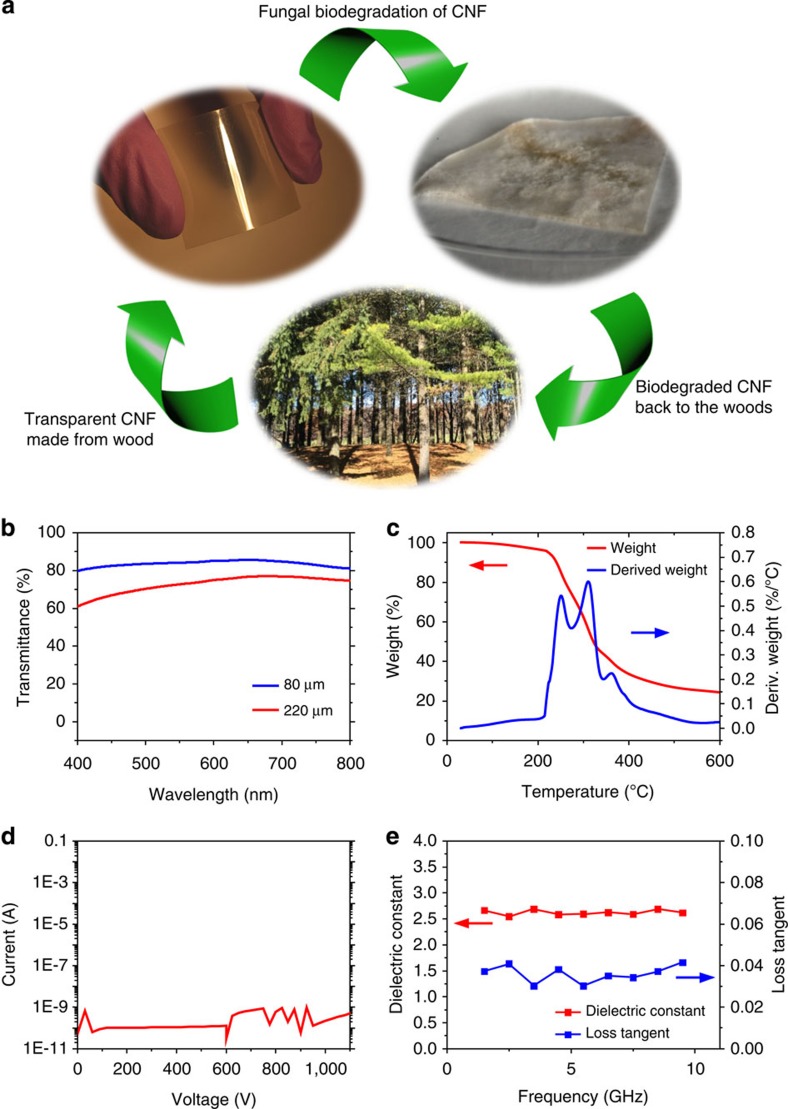
Introduction to cellulose nanofibril (CNF) paper and its basic characteristics as a substrate for electronics. (**a**) An illustration of a likely life cycle of the biobased and biodegradable CNF paper. First, cellulose nanofibrils (CNFs) extracted from the woods is made into CNF paper. The CNF paper can be degraded via fungal biodegradation and sent back to the woods without adverse environmental effects. (**b**) The transmittance curve over a visible spectrum. Blue and red curves show the transmittance of 80-μm and 200-μm thick CNF films, respectively. (**c**) A thermogravimetric (TGA) plot showing the weight change of the CNF film as a function of temperature, along with the first derivative of the curve. The film remains stable up to 213 °C. (**d**) The electrical breakdown characteristics of CNF film. Current is measured while high voltage is applied on both sides of the film. (**e**) Radio frequency characteristics of the CNF film. Dielectric constant (red) and loss tangent (blue) are measured in the frequency range of 0 to 10 GHz using a microstrip waveguide.

Figure 1a presents a likely life cycle of the CNF film where the film is first made from CNFs extracted from the woods, degraded via a fungal biodegradation process on disposal and sent back to the woods without adverse environmental effects. Electronic systems based on such material could significantly facilitate recycling and management of waste streams. Thus, the ecofriendly wood-based CNF substrate is clearly an ideal substitution for electronics that exist today. However, pure CNF film is vulnerable to water^33^. To address this issue, we coated the pure CNF film with a bisphenol A-based epoxy resin. As shown in Supplementary Fig. 1, the epoxy coating increased the contact angle of the CNF film from 28.4° to 74.6°, thereby making the CNF film much more hydrophobic. This treatment allowed for easier handling of the CNF substrate and offered better manufacturing capabilities. Epoxy is a type of thermoset plastic commonly used in electronics packaging materials (for example, electronic molding compounds, as well as underfills) due to its ease of handling, desirable materials properties and relatively low cost. The epoxy coating can also enhance the mechanical properties of the CNF film. Figure 1b–e introduces the unique material properties of epoxy-coated CNF films. As shown in Fig. 1b, the CNF film was transparent, thus making it ideal for certain applications. The transmittance was over 80% for an 80-μm thick CNF film and 60% for a 220-μm thick CNF film over the visible spectrum. Figure 1c presents thermogravimetric analysis (TGA) data showing the weight loss of the epoxy-coated CNF film as a function of temperature as well as the first derivative of the TGA curve. There were three peaks in the differential TGA curve, with the first (213 °C) and third (270 °C) peak corresponding to the decomposition of the CNF and epoxy process, respectively. The middle peak observed at 310 °C was attributed to the overlapping of the CNF and epoxy decomposition peaks. The glass transition temperature (*T*_g_) of the film was measured at 72.8 °C (Supplementary Fig. 2), which was similar to that of polyethylene terephthalate (PET) film, a commonly used substrate for flexible electronics. In addition, the CNF film was strong and flexible enough to allow reversible bending as shown in Supplementary Fig. 3. The flexural modulus of the epoxy-coated CNF film was calculated to be 2.5 GPa, which is comparable to that of PET (1.5 to 2.8 GPa)^34^,^35^,^36^. The electrical properties of the CNF film were also appealing for use with electronics. As presented in Fig. 1d, the CNF film did not undergo an electrical breakdown, even at very high voltages (for example, 1,100 V), which is far beyond the requirement for consumer electronics. Furthermore, because the dielectric and RF properties of the substrate are major aspects to be considered in designing a RF circuit, the RF loss and dielectric constant were extracted using a microstrip waveguide and analysed at high frequencies. In the frequency range from 0 to 10 GHz, the dielectric constant ranged from 2.58 to 2.69, and the loss tangent ranged from 0.0302 to 0.0415, as presented in Fig. 1e. The above characterizations for the first time unveiled the suitability of CNF for high-frequency microwave applications. While the dielectric constant and RF loss values were comparable to those of PET film, the biodegradability property of CNF makes it a superior candidate over PET for addressing the abovementioned environmental impact.

### Fabrication process of GaAs devices on CNF substrates

**Figure 2:**
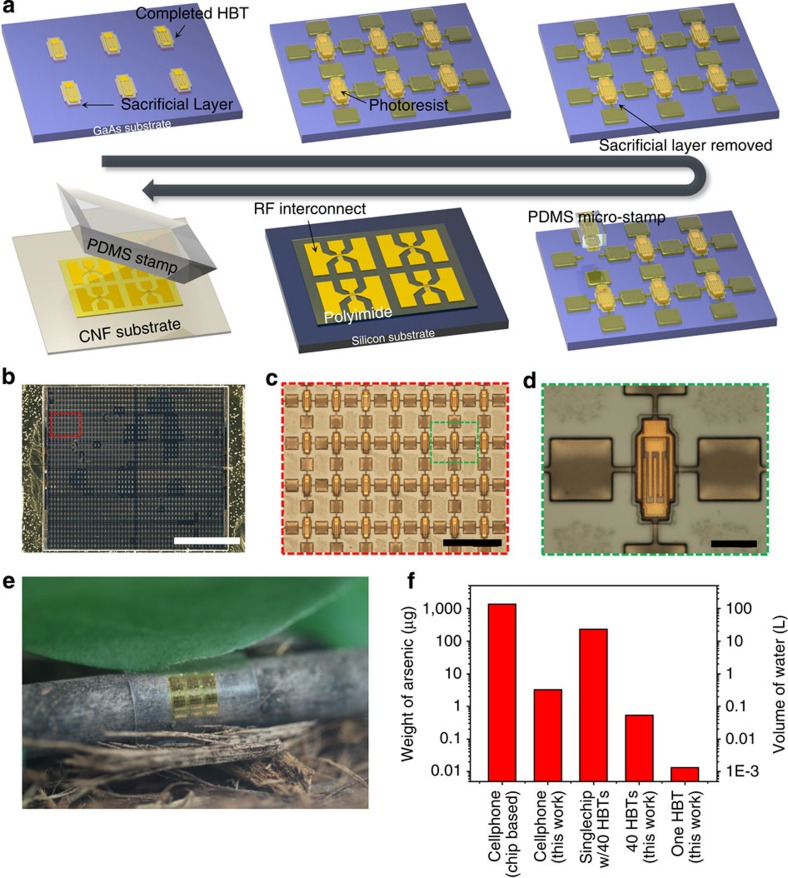
The fabrication process for deterministic assembly of GaAs devices on CNF paper and quantitative analysis on the influence of GaAs to the environment. (**a**) Schematic illustration of the fabrication process of GaInP/GaAs HBTs on a CNF substrate. The HBTs are fabricated on a sacrificial layer grown on a GaAs substrate and released with protective anchors made of photoresists. Each HBT is picked up using a PDMS micro-stamp and printed onto a temporary Si substrate with polyimide as the adhesive. After RF interconnect metallization, the devices are released from the temporary substrate and printed onto a CNF substrate using a PDMS stamp. (**b**) An optical microscopy image showing 1,500 releasable HBTs in a dense array format on a 5 × 6 mm^2^ size GaAs substrate. Scale bar, 2 mm. (**c**) A magnified image of the array. Scale bar, 200 μm. (**d**) An optical image showing a single releasable HBT that is tethered to the substrate with photoresist anchors. Scale bar, 30 μm. (**e**) A photograph of an array of HBTs on a CNF substrate wrapped around a tree stick with a ∼3 mm radius. (**f**) Comparison chart showing the amount of the arsenic corresponding to each type of device/transistor listed as well as the amount of water calculated according to the EPA standard based on the quantity of the arsenic present in these devices/transistors. For a single conventional cell phone, ∼138 l of water is required to satisfy the EPA standard, whereas only 0.32 l is required using our approach. In addition, 23 l is required for a single conventional chip with 40 HBTs, while only 0.054 l is required for the same number of HBTs with our approach. One HBT only requires 0.0013, l of water.

Compared with devices operating at low frequencies (∼MHz) or direct current (DC) levels, microwave (∼GHz) devices are especially difficult to fabricate on foreign substrates, due to the small feature sizes and high temperature processes required for high performance. Here we present, for the first time, methods to fabricate microwave GaAs-based devices on foreign substrates, namely the CNF substrate in this case. It should be noted that today's majority portable gadgets (>85% in cell phones) with wireless communication functions employ GaAs-based microwave devices for their superior high-frequency operation and power handling capabilities. Figure 2a outlines the procedure for manufacturing GaInP/GaAs heterojunction bipolar transistors (HBTs) on a CNF substrate via schematic illustrations. Thin heterojunction epitaxial layers in stacks of n-cap layer (GaAs:Si)/n-emitter layer (GaInP:Si)/p-base layer (GaAs:C)/n-collector layer (GaAs:Si)/n-sub-collector layer (GaAs:Si) were grown on a 500-nm thick sacrificial layer (Al_0.96_Ga_0.04_As) on a GaAs wafer. The fabrication process began by following conventional procedures to fabricate the HBTs (Supplementary Fig. 4), followed by protective anchor patterning using a photoresist (PR). This will protect the devices and allow the devices to be tethered to the substrate after etching away the underlying sacrificial layer using a diluted hydrofluoric acid (HF) solution. Van der Waals contact with a soft elastomer stamp made of polydimethylsiloxane (PDMS) to the device breaks the anchors on all four sides and easily picks up a single device. The devices are transfer printed in deterministic assembly onto a temporary Si substrate using ultrathin polyimide (PI, ∼1 μm) as an adhesive, followed by ground–signal–ground (G–S–G) RF interconnect metallization. PI is an excellent material for GaAs-based devices not only as an adhesive, but also as a passivating material that can suppress the high surface states of GaAs and prevent leakage current^37^. Devices are then released from the temporary substrate and printed onto a CNF substrate using a PDMS stamp (Supplementary Fig. 5). Figure 2b–d presents optical microscopy images of fully formed HBTs on a GaAs substrate that are ready to be picked up. As shown in Fig. 2b, an array of 1,500 releasable HBTs on a 5 × 6 mm^2^ GaAs substrate can be fabricated. The image in Fig. 2e presents an array of HBTs on a CNF substrate wrapped around a tree stick, demonstrating the high flexibility of these electronics.

### Analysis of the influence of GaAs on the environment

The Environmental Protection Agency (EPA) has set the arsenic standard for drinking water at 10 p.p.b.^38^, that is, 10 μg l^−1^. Compared with a typical GaAs MMIC, which only consists of a few HBTs on a large substrate, this pick-and-place method greatly reduces the usage of expensive and hazardous semiconductor materials. Figure 2f presents a quantitative analysis on the amount of arsenic present in the corresponding device/transistor due to the usage of GaAs that may lead to adverse environmental impact. Also shown in Fig. 2f is the amount of water calculated according to the EPA standard based on the amount of arsenic present in these devices/transistors. This analysis shows that a significant amount of clean water can be saved or preserved using our deterministic assembly approach in making the GaAs-based electronics. The weight of arsenic was obtained by converting the measured volume of either conventional GaAs chip or our printed HBTs to weight. As an example, a conventional miniature GaAs HBT-based MMIC with 40 HBTs on a 1.15 × 0.75 mm^2^ large and 100-μm thick substrate^39^ was used as a reference for the comparison. Moreover, our single GaAs HBT with a volume of 5.04 × 10^−6^ mm^3^ was used. Assuming that there are six GaAs HBT-based MMIC chips in a typical cell phone, ∼138 l of water is required at minimum to meet the standards, whereas the same cell phone using our approach only requires 0.32 l of water. For a single conventional chip with 40 HBTs, 22.9 l of water is required, whereas only 0.054 l is required for the 40 HBTs fabricated using our method. This approach is even more advantageous where only a few HBTs are required. For instance, a single conventional chip with 40 HBTs and 20 HBTs would have similar weight because they are typically built on a similarly sized substrate; however, 20 HBTs printed using our approach would weigh exactly half of the 40 HBTs. In fact, a single printed HBT only requires 0.0013, l of water to meet the EPA standard for drinking water.

### Microwave GaAs electronic devices on CNF substrates

**Figure 3:**
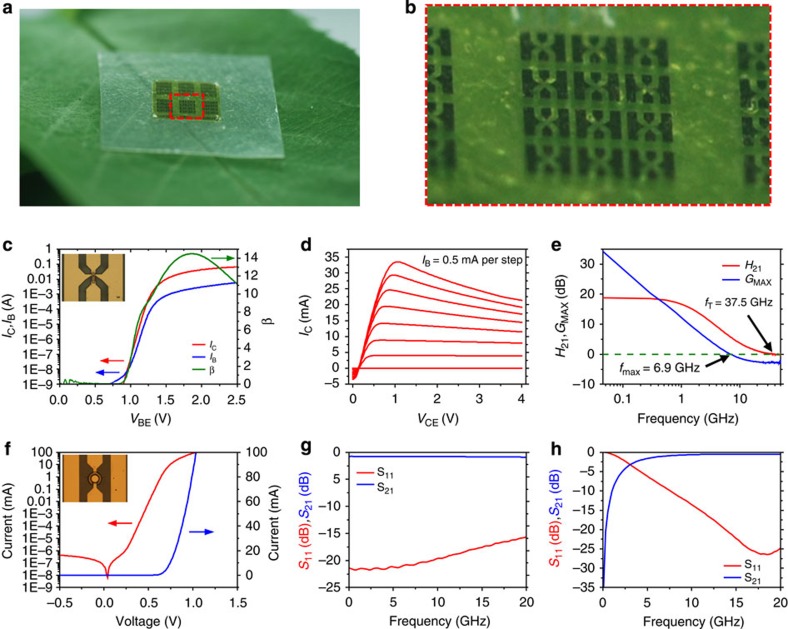
Microwave active GaAs electronic devices on CNF paper. (**a**) Photograph of an array of HBTs on a CNF substrate put on a tree leaf. (**b**) Magnified photograph of the array. (**c**) Gummel plot and *β* plot showing the maximum DC gain of the HBT. The maximum *β* is 14.49. The inset optical image shows one of the HBTs in the array that was measured and characterized. (**d**) *I*_C_ versus *V*_CE_ plot of the HBT plotted at 0.5 mA steps of *I*_B_. (**e**) Current gain (*H*_21_) and power gain (*G*_MAX_) as a function of frequency, with a collector voltage bias of 2 V and a base current bias of 2 mA. (**f**) Current versus voltage plot of the Schottky diode on a CNF substrate. The red curve shows the logarithmic scale and the blue curve shows the linear scale. The inset optical image shows the diode transferred onto a CNF substrate with G–S–G interconnects. (**g**) Measured S_11_ (red) and S_21_ (blue) plotted against frequency under a forward current bias of 10 mA. (**h**) Measured S_11_ (red) and S_21_ (blue) plotted against frequency under a reverse voltage bias of −0.5 V.

Figure 3c–e shows the electrical properties of a single finger (2 × 20 μm^2^) non-self-aligned HBT on a CNF substrate, which is optically shown in Fig. 3a,b. The inset image of Fig. 3c shows an optical microscopy image of the device that was measured. The Gummel plot presented in Fig. 3c reflects collector and base electric current, *I*_C_ and *I*_B_, against base-emitter voltage, *V*_BE_ with zero *V*_BC_ bias. The common-emitter current gain curve under zero *V*_BC_ bias is shown in green in Fig. 3c, which indicates that the *β* had its maximum value of 14.49 at a *V*_BE_ of 1.86 V. Under an extreme bending condition (that is, at a bending radius of 2.5 mm), the maximum *β* value decreased slightly to 13.64 (Supplementary Fig. 6). The *I*_C_ versus *V*_CE_ curve is presented in Fig. 3d. The positive *V*_CEOFFSET_ value of 0.14 V is due to the single heterojunction structure of our HBT where the offset comes from the difference in bandgap between the emitter (GaInP) and the base (GaAs). The decaying collector current observed as *V*_CE_ was increased at high base current is attributed to poor thermal dissipation as the thermal conductivity of the underlying CNF substrate (*κ*=1.0 W m^−1^ K^−1^)^40^ was lower than that of a typical GaAs substrate (*κ*=56 W m^−1^ K^−1^)^41^. The RF performances of the HBT were analysed from the measured scattering (S) parameters from 0.045 to 50 GHz (Supplementary Fig. 7). Open and short patterns of the probing pads on the CNF substrate were used to subtract the effect of parasitic inductances and capacitances of the pad. Figure 3e presents the current gain (*H*_21_) and power gain (*G*_MAX_) against frequency for the device under a bias of *V*_C_=2 V and *I*_B_=2 mA. A high cut-off frequency (*f*_T_) of 37.5 GHz and a maximum oscillation frequency (*f*_max_) of 6.9 GHz were obtained. The relatively low *f*_max_ of 6.9 GHz was attributed to the non-self-aligned structure of the HBT where large emitter-base spacing (2 μm) introduced high base resistance, causing the *f*_max_ to drop^42^. These outstanding RF results further prove the suitability of CNF for microwave applications. Although we observed a decay of current at increasing voltages due to the relatively low thermal conductivity of the CNF film, the frequency responses of the HBT were sufficiently high to be used as practical amplifiers in mobile devices where the cellular frequency is in the range of 800 to 2,500 MHz. By incorporating materials with high thermal conductivities, such as boron nitride or diamond nanoparticles into the CNF film, the device performance can be further improved.

Schottky diodes based on GaAs are commonly used in high speed communication systems such as mixers and rectifiers. Same fabrication techniques with minor changes, as described in Fig. 2a can be implemented to fabricate high-performance Schottky diodes. Similar to the HBTs, nearly 1,200 Schottky diodes with high yield can be fabricated on a 5 × 6 mm^2^ GaAs substrate. Figure 3f (with an inset image showing the measured diode) presents the DC performance of the diode measured on a CNF substrate, where an ideal Schottky behaviour with a low turn-on voltage of 0.7 V was obtained. A logarithmic plot (shown in red) of the data shows a good ideality factor of 1.058. Figure 3g,h presents the measured S-parameters of the diode at forward bias and reverse bias, respectively (polar plots and Smith charts are shown in Supplementary Fig. 8). At a forward current bias of 10 mA (*V*=740.6 mV), the insertion loss (S_21_) was only −1 dB at 20 GHz, making it suitable for RF applications. At a reverse voltage bias of −0.5 V (*I*=−414.1 pA), the insertion loss (S_21_) reached −2 dB at 4.3 GHz. The low resistance obtained under reverse bias at high frequencies shows that these diodes can perform with high switching speeds in microwave circuits.

**Figure 4:**
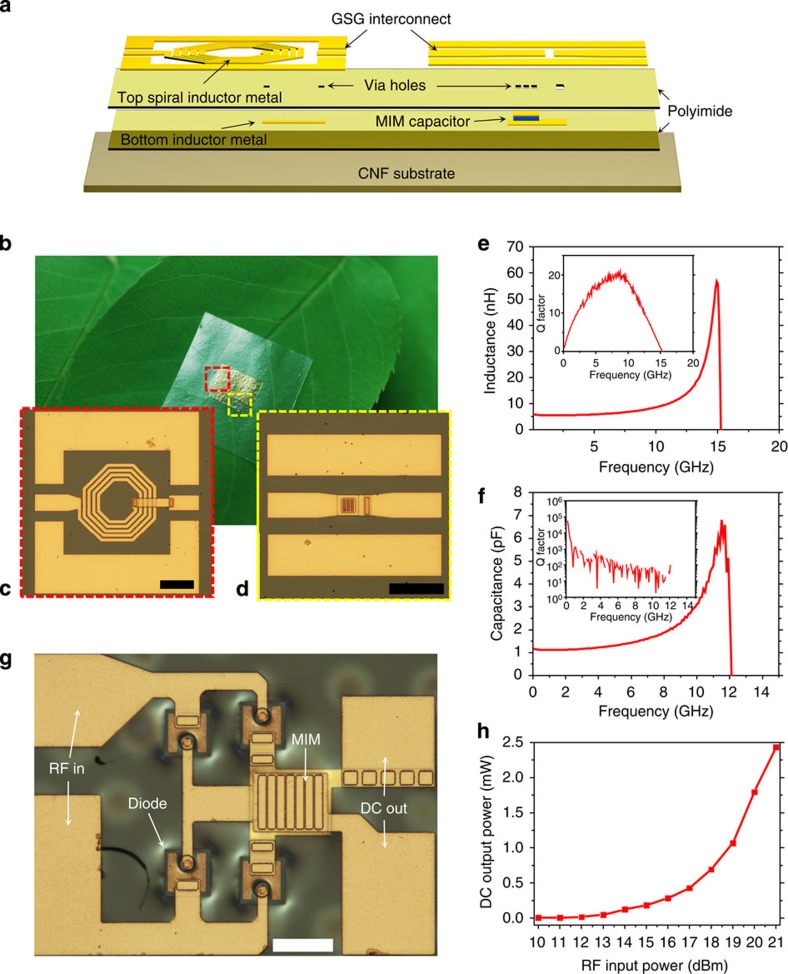
Microwave passive elements and integrated circuit on CNF paper. (**a**) An exploded view schematic illustration of the inductor and capacitor on a CNF substrate. (**b**) Array of inductors and capacitors on a CNF substrate put on a tree leaf. (**c**) Optical image of the measured 4.5 turn inductor. Scale bar, 100 μm. (**d**) Optical image of the measured MIM capacitor. Scale bar, 100 μm. (**e**) Inductance plotted against frequency with an inset plot showing the inductor *Q* factor as a function of frequency. (**f**) Capacitance plotted against frequency with an inset plot showing the capacitor *Q* factor as a function of frequency. (**g**) An optical microscopy image of a full bridge rectifier built on a CNF paper. Here the microwave Schottky diodes and an MIM capacitor were integrated. Scale bar, 50 μm. (**h**) Measured rectified DC output power of the rectifier while applying RF input power from 10 to 21 dBm at 5.8 GHz.

Passive elements are crucial components that are used for various purposes, such as RF chokes and impedance matching networks in RF circuits. To demonstrate the full capability of the CNF substrate for microwave circuit application, simple metal–insulator–metal (MIM) capacitors and spiral inductors were fabricated on a CNF substrate. Figure 4a presents the structure of the two passive elements on a CNF substrate with schematic illustrations. Bottom inductor metal and MIM capacitors, with 200 nm of TiO_2_ as the dielectric material, were deposited on a releasable thin PI (∼1 μm) sheet spin casted on a temporary Si substrate. Another PI layer served as via holes during the subsequent metallization step for the G–S–G RF interconnects. The finished passive components were then released from the temporary substrate and transfer printed onto the CNF substrate. Figure 4b shows a photograph of the inductors and capacitors on a CNF substrate placed on a tree leaf. Figure 4c,d shows the optical microscopy images of the measured inductor and capacitor, respectively. Measured S-parameters are plotted in Supplementary Fig. 9. The inductance of the 4.5 turn inductor versus frequency is plotted in Fig. 4e. The width of the metal line of this inductor was 10 μm and the spacing between the adjacent metal lines was 5 μm. A constant inductance of ∼6 nH was obtained up to ∼8 GHz, with a self-resonant frequency (*f*_res_) of 15.1 GHz. A peak *Q* value of ∼20 was obtained at 8 GHz as shown in the inset image of Fig. 4e. Figure 4f plots capacitance against frequency for a 30 × 30 μm^2^ MIM capacitor with *Q* factor plotted in the inset image. A constant capacitance of ∼1.3 pF was measured up to 6 GHz, with a *f*_res_ of 12.1 GHz. Such high *Q* and *f*_res_ values obtained at a broad frequency range suggest that these inductors and capacitors are applicable for high speed RF integrated circuits, in conjunction with the microwave devices, on CNF substrates. To evaluate the printed microwave devices on a CNF substrate in an application, four microwave GaAs-based Schottky diodes and an MIM capacitor were combined into a simple integrated circuit to form a full bridge rectifier, as optically shown in Fig. 4g with its circuit diagram shown in Supplementary Fig. 10. The rectification behaviour of RF-to-DC conversion at 5.8 GHz is shown in Fig. 4h. This frequency is one of the popular frequencies in wireless local area network, commonly used in high speed Wi-Fi systems. As shown in the plot, the rectifier can rectify a 21 dBm input signal to an output power of 2.43 mW. The ability to rectify such high-frequency signals can be attributed to the excellent electron mobility of GaAs and the low turn-on voltage of the Schottky diodes. With an appropriate matching network, the rectification ratio is expected to increase drastically by enhancing the reflection loss of the circuit. S_11_ of the rectifier is shown in Supplementary Fig. 11.

### Si-based digital electronics on CNF substrates

**Figure 5:**
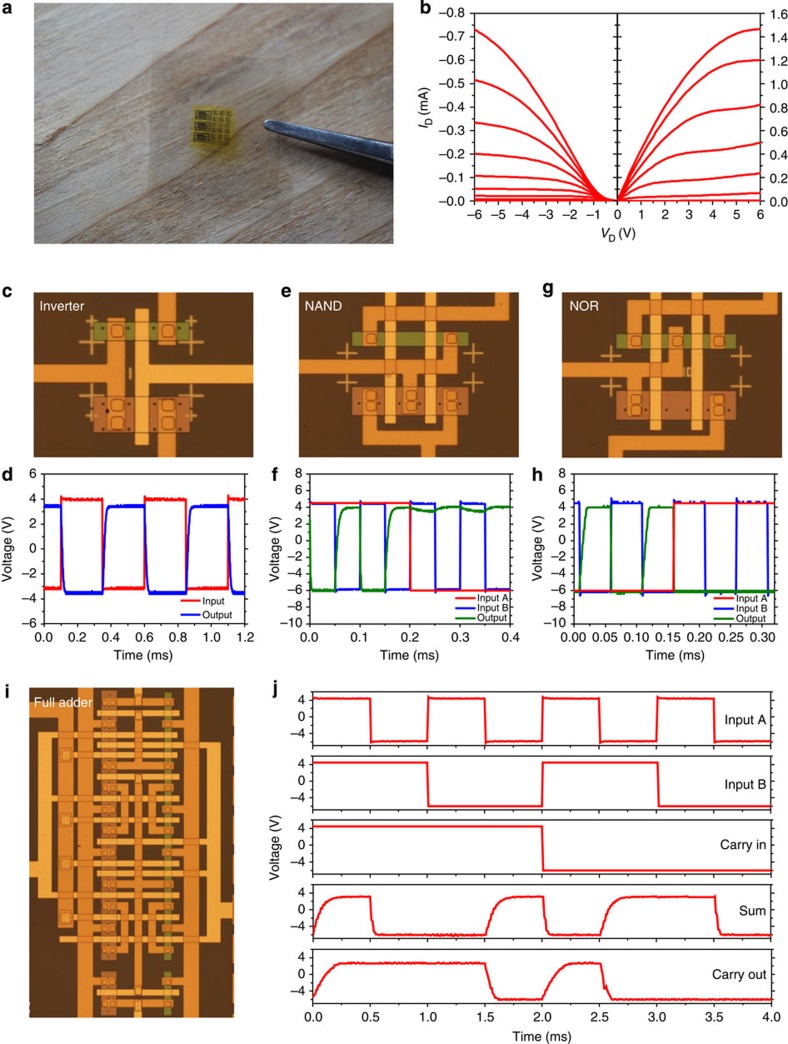
Digital electronics on CNF paper. (**a**) A photograph of CNF paper with digital electronics. (**b**) *I*_D_ versus *V*_D_ plot of a p-type MOSFET (left) and an n-type MOSFET (right) at *V*_G_ steps of 1 V. (**c**) An optical microscopy image of an inverter. (**d**) Input–output characteristics of the inverter. (**e**) An optical microscopy image of a NAND gate. (**f**) Input–output characteristics of the NAND gate. (**g**) An optical microscopy image of a NOR gate. (**h**) Input–output characteristics of the NOR gate. (**i**) An optical microscopy image of a full adder. The adder consists of 28 transistors. (**j**) Characteristics of the full adder: Input A, Input B, Carry In, Sum and Carry Out are shown in descending order.

In addition to microwave electronics that allow wireless communication for mobile electronic devices, digital circuits are also important components that are dominant in most electronic devices as microprocessors and controllers. Figure 5 summarizes a set of digital logic circuitries on a CNF substrate using Si-based complementary metal–oxide–semiconductor (CMOS) devices. Figure 5a shows the completed digital circuits on a CNF substrate, which includes ‘universal' logic gates (Inverter, NOR gate and NAND gate) and a full adder. The fabrication was done by separately printing Si nanomembrane-based p-type metal–oxide–semiconductor field-effect transistors (MOSFETs) and n-type MOSFETs onto a PI-coated temporary Si substrate, followed by deposition of gate oxides and metal interconnects for making CMOS-based digital circuits. A sequence of schematic illustrations describing the fabrication process is shown in Supplementary Fig. 12. Figure 5b presents the current–voltage characteristics of the p-type MOSFET (left) and n-type MOSFET (right). Figure 5c shows an optical image of the CMOS inverter. As presented in Fig. 5d, the inverter exhibits a good input and output relationship. A further modelling of these CMOS transistors established NOR and NAND logic gates, which are optically shown in Fig. 5e,g, respectively. The input and output relationships of the NOR and NAND gates are shown in Fig. 5f,h, respectively. The inputs and outputs can be seen as well-defined ‘0's and ‘1's. All of these components can be used together to yield a simple integrated circuit on a CNF substrate. As an example, a full adder, which is highly scalable and useful in many cascaded circuits, was designed and fabricated on a CNF substrate, as optically shown in Fig. 5i. This full adder is a mirror full adder, which consisted of 28 transistors with 4 of them used for inverter construction. As presented in Fig. 5j, the two single bit outputs (SUM and Carry Out) had a 0.2 ms switching delay when responding to the three single bit inputs (Input A, Input B and Carry In). This made the full adder work at a frequency of up to 5 kHz.

### Fungal biodegradation tests of the CNF-based electronics

**Figure 6:**
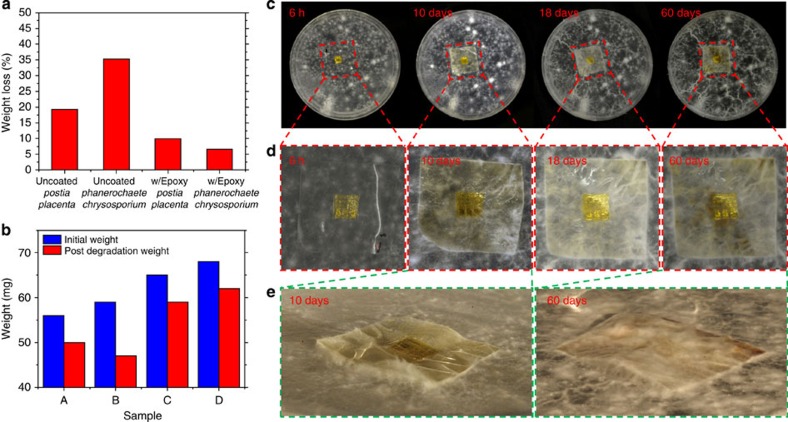
Fungal biodegradation tests of the CNF-based electronics. (**a**) Fungal biodegradation tests of two types of CNF films. The left two bars show the per cent weight loss for uncoated pure CNF films. The right two bars show per cent weight loss for epoxy-coated CNF films. The tests suggest that *Postia placenta* degrades the uncoated CNF films slower than *Phanerochaete chrysosporium*; however, *Postia placenta* degrades the epoxy-coated CNF films faster than *Phanerochaete chrysosporium*. (**b**) Fungal biodegradation tests of digital electronics printed on top of the epoxy-coated CNF films. Four samples were degraded with *Postia placenta*. (**c**) A series of photographs taken at 6 h, 10 days, 18 days and 60 days after starting the degradation process. (**d**) A series of magnified photographs of the CNF-based electronics during the degradation process. (**e**) Tilted view photograph of the CNF-based electronics after 10 days and 60 days of degradation. The fungus fully covers the film after 60 days.

As presented in Figs 2, 3, 4, 5, all types of electronic systems required for building an electronic device can be realized on a CNF paper. To prove the concept of biodegrading electronic devices and to close the cycling loop that is shown in Fig. 1a, one of the electronics that we have presented here was subjected to a fungal degradation test. Figure 6 summarizes a sequence of fungal degradation tests on CNF-based electronic devices. First, two different types of decay fungi, brown rot fungus *Postia placenta* and white rot fungus *Phanerochaete chrysosporium*, were considered and tested on the pure CNF substrate and on the epoxy-coated CNF substrate, without any electronics printed on them. Figure 6a presents the average weight loss percentages of these CNF-based films after 28 days. For each degradation test, five identical samples were degraded under the same conditions. Pure CNF samples showed a larger average weight loss (*Postia placenta*: 19.20%, *Phanerochaete chrysosporium*: 35.20%) compared with the epoxy-coated samples. While *P. placenta* induced a slower degradation rate for pure CNF film, it caused a faster degradation for the epoxy-coated CNF film (*P. placenta*: 9.96%, *P. chrysosporium*: 6.60%) in comparison with *P. chrysosporium*. Therefore, *P. placenta* was chosen as the decaying agent for our CNF-based electronics that consisted of the epoxy-coated CNF film. The amount of epoxy in the epoxy-coated CNF film was 9.6% by weight. Figure 6b shows the weight loss result of digital electronics on CNF substrates after *P. placenta* decaying for 84 days. Four replicas were made, and on average, the weight loss percentage was 12.25%, with a s.d. of 5.43%, suggesting that the CNF film will fully degrade after an extended period of time. Figure 6c–e shows the images of the decaying process of an epoxy-coated CNF substrate with digital electronics against *P. placenta*. Photos were taken after 6 h, 10 days, 18 days and 60 days as shown in Fig. 6c,d. As presented in Fig. 6e, the fungi started to partially cover the sample after 10 days, and fully covered the sample after 60 days. Once degraded, the leftover electronics portion, which is encapsulated in PI, can be collected to be further decomposed and recycled. Although PI can deteriorate with certain fungi, the degradation process is extremely slow compared with CNF, and because PI is generally non-permeable to water or solvents it can be used to protect against any leakage of materials to the environment^43^.

## Discussion

In summary, the feasibility of a sustainable, green chip concept that is applicable in both microwave and digital electronics, by strategically combining the minimum use of expensive, environmentally toxic semiconductor materials and the employment of microwave compatible, biodegradable CNF as substitutional substrates, was established. The demonstrated excellent performance GaAs-based HBTs and Schottky diodes, passive inductors and capacitors, and Si-based CMOS digital devices, ‘universal' logic gates and integrated full adders on CNF substrates, which are essential components in most typical electronic systems, share common fabrication techniques that can be easily integrated together. The combination of all of these thin-film form components into large scale integrated circuits on CNF substrates would provide ways to make many types of fully functional and ecofriendly electronics that could help reduce the accumulation of the massive amounts of persistent electronic waste disposed daily and dramatically reduce the consumption of non-renewable natural resource. At the system level, there have been on-going efforts to reduce the use of toxic materials like lead, mercury and arsenic that may be present in components such as batteries and displays^44^,^45^,^46^. These combined efforts will lead to a new generation of more ecofriendly electronics.

## Methods

### Preparation of CNF paper

The tetramethylpiperidine-1-oxy (TEMPO)-oxidized CNFs were refined in a microfluidizer processor (Microfluidics, Newton, MA), followed by filtering (Millipore Corporation, USA) under air pressure (0.55 MPa) with polytetrafluoroethylene membranes that have 0.1 μm pore sizes. Subsequently, the filter cake was separated from the membrane and sandwiched between layers of waxy coated paper, filter paper and caul plates at room temperature for drying, followed by further drying in an oven at 60 °C for several hours. The dried CNF film was then coated with a bisphenol A-based epoxy resin (56:24:24 mixture of low viscosity epoxy resin, flexible epoxy resin and amine-based curing agent, Dow Chemical Company) and pressed at 130 °C for 10 min under a pressure of 100 psi.

### Characterization of CNF paper

A contact angle goniometer (OCA 15/20, Future Digital Scientific Corp., USA) was used for the water contact angle measurements at ambient temperature. The volume of the water droplet was fixed at 4.0 μl, and the contact angle was determined 1 s after the water droplet was deposited on the surface of the CNF film. Three point flexural tests were conducted using a dynamic mechanical analyser (TA Instruments RSA III, USA). Rectangular epoxy-coated CNF film (with a length of 40 mm, a width of 13 mm and a thickness of 0.2 mm) was used for the flexural tests. The maximum flexural deflection was set at 5 mm for the tests. To measure transmittance, CNF film with a thickness of either 80 μm or 200 μm was loaded onto a spectrophotometer (5000 UV–vis-NIR, Cary). The system was set to transmission mode and the transmittance was recorded every 1 nm throughout the spectrum from 400 to 800 nm. The thermal stability of the epoxy-coated CNF films were characterized via TGA using a TGA/Q50 thermal analyzer (TA Instruments, DE, USA). Approximately 10 mg of the CNF films were heated from 30 to 600 °C at a heating rate of 10 °C min^−1^ in an N_2_ atmosphere. Differential scanning calorimetry (DSC) was performed in an N_2_ atmosphere using a DSC thermal analyzer (Auto Q20, TA Instruments) from 0 to 160 °C at a heating rate of 5 °C min^−1^ and a N_2_ flow rate of 20 ml min^−1^. The sample (∼8.0 mg) was stored in a sealed aluminium pan. To measure the electrical breakdown characteristics, metal (Ti/Au, 10/200 nm) was evaporated on both sides of a 200-μm thick CNF film via a shadow mask, with a pad of 300 μm in diameter. High voltage was applied using a voltage source (2410 High-Voltage Source Meter, Keithley) through standard DC probing while the current was monitored. To measure the dielectric constant and loss tangent, the microstrip transmission line-approximation-method was used. A square CNF film with an area of 17.64 cm^2^ was attached with a copper film as the ground on the back side, and a 6-mm wide copper tape as the transmission line on the centre of the top side. S-parameters were collected through the SMA connectors as the RF signal was transmitted through the microstrip transmission line. The dielectric constant and loss tangent of the CNF film were then extracted according to the small signal circuit approximation.

### Fabrication of high speed GaAs HBTs

The fabrication process began by depositing emitter finger metals (Pd/Ge/Au, 30/40/200 nm) using an electron-beam evaporator via a PR (AZ5214) lift-off process, followed by inductively coupled-reactive ion etching (ICP-RIE, BCl_3_/Ar=10/5 sccm, pressure=2 mTorr, plasma power=50 W, inductor power=500 W) of the cap and emitter layer. Another PR lift-off process to deposit base metal fingers (Ti/Pt/Au=10/30/200 nm) and ICP-RIE etching using SiO_2_ (800 nm) as a hard mask were carried out next to expose the sub-collector layer. After depositing collector metal fingers (Pd/Ge/Au=30/40/200 nm), the sample was annealed at 450 °C for 30 s in ambient forming gas (H_2_/N_2_=5/95%) in a rapid thermal anneal (RTA) system for ohmic contact formation. Isolation of individual devices was done using ICP-RIE to etch the sub-collector layer and the underlying sacrificial layer. Protective anchors were patterned by spin casting a thick (∼7.0 μm) PR layer (Megaposit SPR220, Rohm and Haas Electronic Materials) at 4,000 r.p.m. for 30 s, soft baked at 110 °C for 120 s, exposed to ultraviolet light at a dose of 500 mJ cm^−2^, developed (MF-24A) for 120 s and hard baked at 110 °C for 10 min. The AlGaAs sacrificial layer was undercut etched using diluted HF (1:100=deionized water: 49% HF) for 3 h.

### Fabrication of GaAs Schottky diodes

A hard mask of SiO_2_ (800 nm) was deposited via a lift-off process, followed by ICP-RIE etching of an n^−^ GaAs layer to reach an n^+^ GaAs layer. Cathode metal (Pd/Ge/Au=30/40/200 nm) was deposited next via a lift-off process and annealed in RTA (same conditions as the HBT RTA process) for ohmic contact formation. A Schottky metal (Ti/Pt/Au=10/30/200 nm) was deposited on an n^−^ GaAs layer for anode contact, followed by an ICP-RIE isolation process, patterning of the protective anchor and sacrificial layer etching using the same procedures used for HBTs.

### Preparation of the micro-stamp

A pattern of negative PR (SU8 50, Microchem, 100 μm) on a Si substrate was prepared for PDMS (Slygard 184, Dow Corning, 10:1 mixture of pre-polymer to curing agent) molding of an 80 × 80 μm^2^ elastomer micro-stamp for selective transfer printing of the devices.

### Fabrication of GaAs devices on a temporary substrate

On a Si substrate, a thin layer of sacrificial polymer, that is, polymethyl methacrylate (950 PMMA A2, Microchem, 60 nm) was spin casted, followed by hard baking at 180 °C for 3 min. A thin sheet of PI (Sigma-Aldrich, ∼1 μm) was spin casted at 5,500 r.p.m. for 60 s on the top, followed by soft bake at 80 °C for 25 s to create adhesion. Using a micro-stamp mounted on a modified mask aligner (MJB-3 aligner, Karl Suss), an HBT or a Schottky diode was transfer printed on the PI adhesive and hard baked at 130 °C for 3 min. A quick spray of acetone removed the protective anchor on the device, but left the PI undamaged. Another thin sheet of PI (∼1 μm) was spin casted, followed by soft bake at 150 °C for 5 min and hard bake at 300 °C for 1 h in a vacuum oven. Copper (100 nm) was deposited using an electron-beam evaporator with a lift-off process to serve as a hard mask to open via holes for the device contacts, followed by reactive ion plasma etching (RIE, CF_4_/O_2_=2/40 sccm, pressure=300 mTorr, power=200 W) of PI and wet etching of a copper mask (Copper Etch APS-100). G–S–G RF pads (Ti/Cu/Ti/Au=10/1,800/10/200 nm) were deposited via a lift-off process for DC and RF characterization of the devices.

### Fabrication of passive devices on a temporary substrate

On a Si substrate with a PMMA sacrificial layer and a thin sheet of PI, a bottom inductor metal and capacitor metal (Ti/Au=10/300 nm) were deposited via a lift-off process. Photolithography patterning on the bottom capacitor metal defined the capacitor size, where the dielectric material (TiO_2_=200 nm) and top capacitor metal (Ti/Au=10/300 nm) were deposited. With PI (∼1 μm) spin casted, copper (100 nm) served as a hard mask to open via holes for a top spiral inductor metal and a G–S–G interconnect (Ti/Cu/Ti/Au=10/1,800/10/200 nm) deposited via a lift-off process.

### Fabrication of microwave rectifier on a temporary substrate

Both Schottky diodes and MIM capacitors were integrated together by utilizing the same fabrication processes described above for these two types of devices.

### Fabrication of digital electronics on a temporary substrate

n-type and p-type active regions for CMOS were prepared separately on the p-type (4 × 10^15 ^cm^−3^) and n-type (5 × 10^14 ^cm^−3^) Si-on-insulator (SOI) wafers. Before ion implantation, 20 nm of thermal oxides (Tystar Oxidation Furnace) were grown on both n- and p-type SOIs for screen oxides. Uniform ion implantation was followed to slightly raise the doping concentration of the active region to minimize channel resistances and adjust the threshold voltage of the MOSFET. A short period of thermal annealing in a furnace was applied to recover the defects generated from implantation and activate the dopants. Heavy ion implantation was applied on the photolithography pre-defined source and drain region. Implantation details are listed in Supplementary Table 1. After a second thermal annealing in the furnace, active regions on the SOI wafers were isolated using reactive plasma etching (Unaxis 790). The SOI wafers were then placed in a diluted HF (1:10) solution to etch the sacrificial buried oxide layer and release the membrane. PI (∼1 μm) was spin casted and cured on a 60-nm thick PMMA-coated Si wafer. A soft bake of 1 min at 100 °C dried out the solvent while maintaining the adhesive surface. Individually released membranes from each type of the SOIs were aligned and transferred separately onto the PI using a PDMS stamp mounted on a modified mask aligner, followed by hard bake at 300 °C for 1 h in a vacuum oven. A standard source/drain metal pad, dielectric layer, via hole openings and gate process for CMOS fabrication were processed on a temporary substrate (Supplementary Fig. 12). A second PI layer was coated on the surface for passivation and protection followed by via hole etching for the measurement pads.

### Transfer printing electronics on a CNF substrate

The PI encapsulated devices (HBT, Schottky diode, inductor and capacitor, and digital electronics) on temporary Si substrates were boiled in acetone at 200 °C for 2 h to remove the underlying sacrificial layer (PMMA). A large PDMS elastomer stamp was used to transfer print the finished devices onto the CNF substrate with a thin layer of polymer (SU8 2000.5, Microchem, 500 nm) as the adhesive layer to ensure good bonding between the CNF substrate and the transferred devices.

### Measurement and analysis

An Agilent N5225A PNA Network Analyzer was used to measure the S-parameter of the microstrip transmission line based on a CNF film. For the devices, the DC measurements were performed using an HP 4155B Semiconductor Parameter Analyzer, and RF measurements were performed using an Agilent E8364A PNA Series Network Analyzer. The measurement set-up of the network analyser was calibrated to the G–S–G probe tips using a standard Short-Open-Load-Thru (SOLT) calibration kit. HP 8350B Sweep Oscillator and 83592B RF Plug-in systems were used to provide RF power to the rectifier. The DC output signals were measured using a Rigol DS1102E oscilloscope with a 10 Ω resistor as the load. The S-parameters obtained from the RF measurements were analysed using the Advanced Design System (ADS) software.

### Fungal biodegradation tests of CNF-based electronics

To prepare for a fungal degradation test, the two decay fungi, *P. placenta* (Fr.) M.Lars. and Lomb. (MAD 698) and *P. chrysosporium* (ME461) were grown and maintained on 2% malt agar (DifCo, Detroit, MI) in petri dishes (15 × 100 mm). Inoculum was incubated at 27 °C in a 70% relative humidity (RH) room for 2 weeks to obtain confluent growth on petri dishes. Meanwhile, the CNF films or CNF-based electronics went through a 24 h cleaning process in a propylene gas chamber. The cleaned samples were then laid on agar plates containing the confluent fungal growth according to American Wood Preserver's Association E-10-06 standard. Evaluations were observed at 6 h, 10 days, 18 days and 60 days for fungal growth on specimens; photographic records were obtained. Photographs were taken at time zero as a control.

### Weight loss determination of degraded CNF substrate

Pure CNF and epoxy-coated CNF substrate specimens either with or without electronics were preconditioned in a 27 °C, 65% RH conditioning room for 2 weeks. The weights were measured and recorded as the initial weight. Thereafter, specimens were loaded in petri dishes, allowing fungal growth and degradation in a 27 °C, 70% RH room. At the end of 84 days (28 days for samples without electronics), specimens were harvested, fungal mycelia was brushed off, air dried for 48 h and reconditioned for 14 days. Weights were then measured and recorded as post degradation weight. Weight losses were then calculated and determined.

## Additional information

**How to cite this article:** Jung, Y.H. *et al*. High-performance green flexible electronics based on biodegradable cellulose nanofibril paper. *Nat. Commun.* 6:7170 doi: 10.1038/ncomms8170 (2015).

## Supplementary Material

Supplementary Information Supplementary Figures 1-12 and Supplementary Table 1
