# Supplementary material for: High-performance green flexible electronics based on biodegradable cellulose nanofibril paper
Source: Nat Commun. 2015 May 26;6:7170. doi: 10.1038/ncomms8170 (PMC4455139; doi:10.1038/ncomms8170)
Supplement: Supplementary Information — Supplementary Figures 1-12 and Supplementary Table 1 [file ncomms8170-s1.pdf]

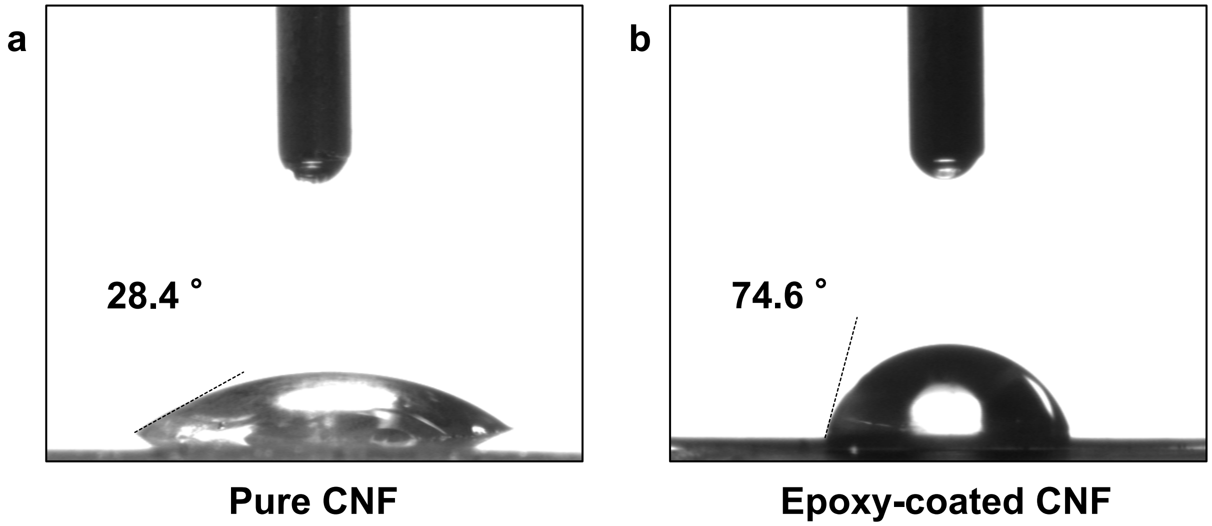

Supplementary Figure 1 | Comparison of the contact angles of a water droplet on a pure CNF film and an epoxy-coated CNF film. The contact angle was (a) 28.4° for the pure CNF paper at t = 1 s and (b) 74.6° for the epoxy-coated CNF paper at t = 1 s.

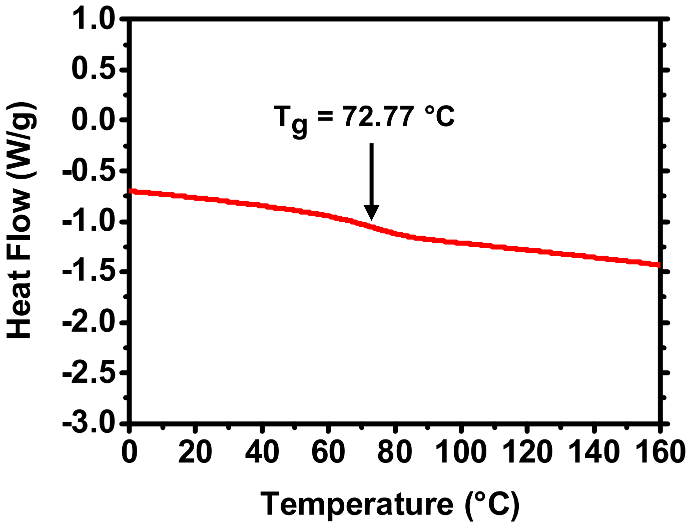

Supplementary Figure 2 | The differential scanning calorimetry (DSC) curve of the epoxy coated CNF film obtained during the second heating cycle.

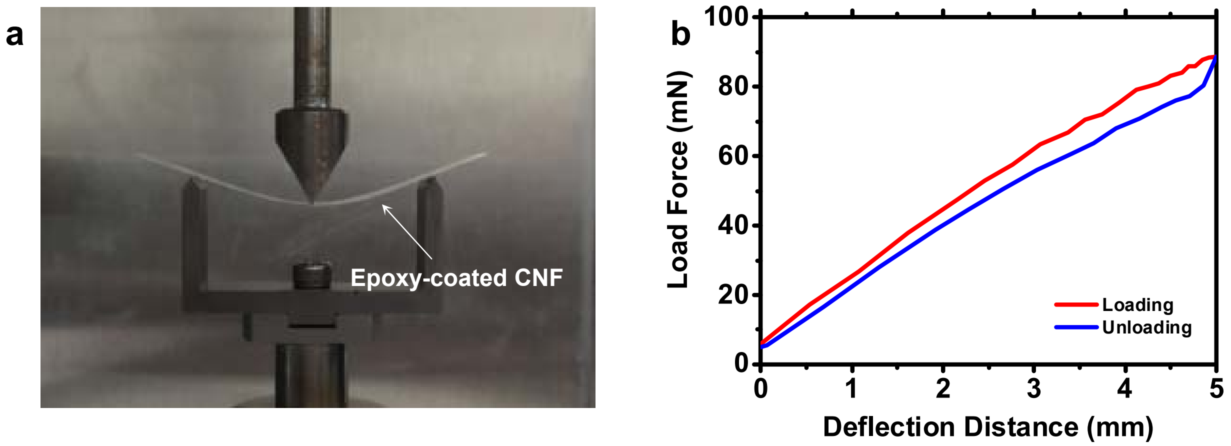

Supplementary Figure 3 | Load-deflection test of the epoxy-coated CNF film using a dynamic mechanical analyzer. (a) Measurement set-up for testing the load-deflection. (b) Measured load force in relation to deflection distance during loading (red) and unloading (blue). The film is reversible back to its flat state after unloading.

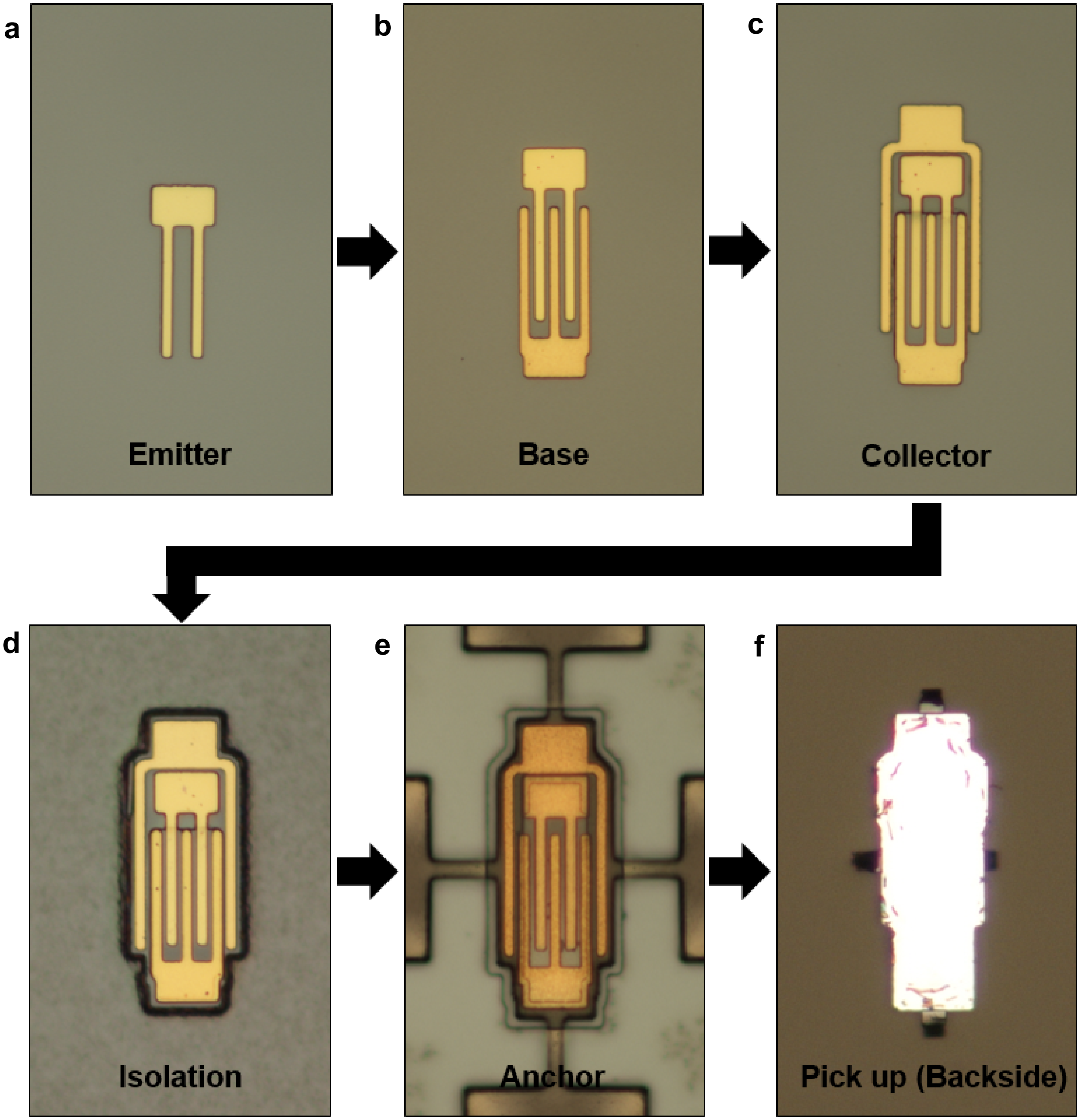

Supplementary Figure 4 | Illustration of the fabrication steps of GaInP/GaAs HBT via a sequence of optical images. (a) Emitter metal is deposited on a GaAs cap layer. (b) Emitter layer is plasma etched and base metal is deposited. (c) After base mesa etching, the collector metal is deposited. (d) Isolation of the HBT is done by plasma etching. (e) Protective anchor patterning on the HBT. (f) HBT is picked up using a PDMS stamp. The image shows the backside of the HBT.

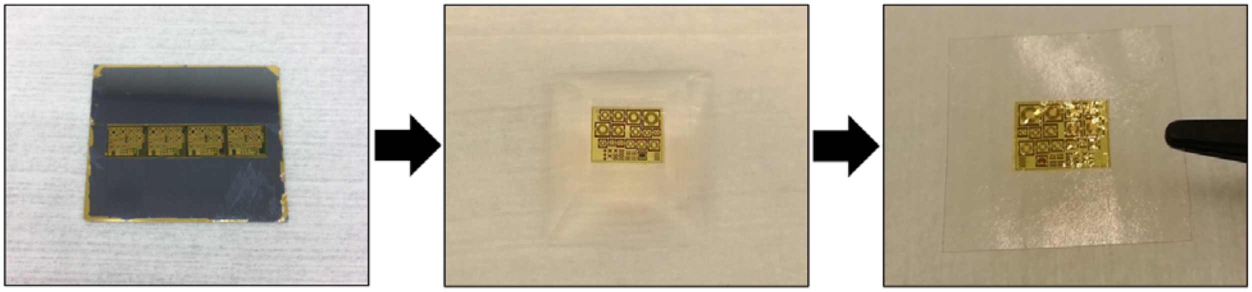

Supplementary Figure 5 | A sequence of optical images demonstrating the transfer process of microwave electronics on a CNF substrate. Here, the transfer process of passive elements is shown as an example. The passive elements are fabricated on a temporary Si substrate (left), picked up using a PDMS stamp (middle), and then transfer printed onto a CNF substrate (right).

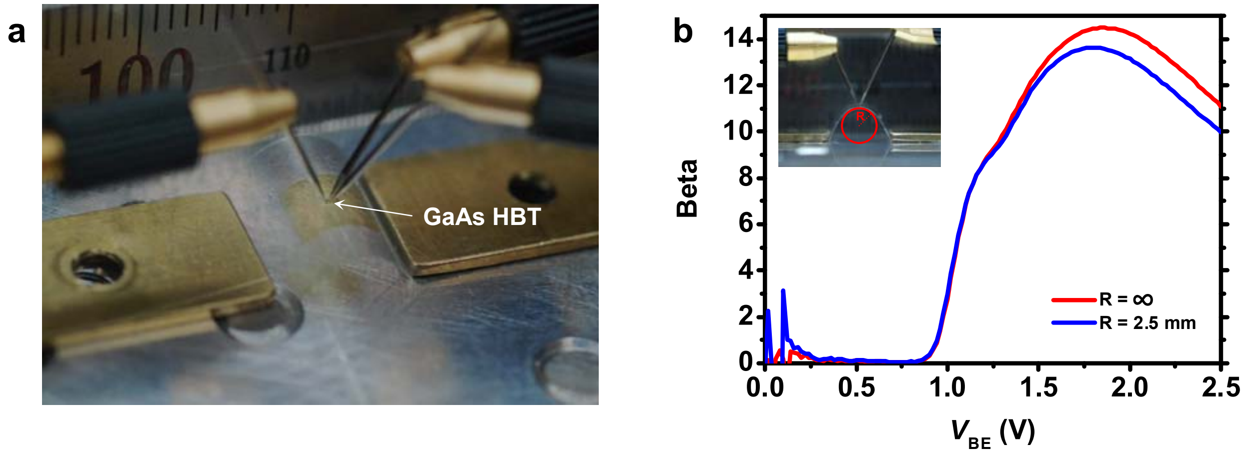

Supplementary Figure 6 | Effect of bending on the Beta gain of GaAs HBT. (a) Measurement set-up for testing the GaAs HBT under bending. (b) Comparison of the Beta plot for GaAs HBT when the CNF substrate was flat (red) or bent (with a radius of curvature of 2.5 mm) (blue).

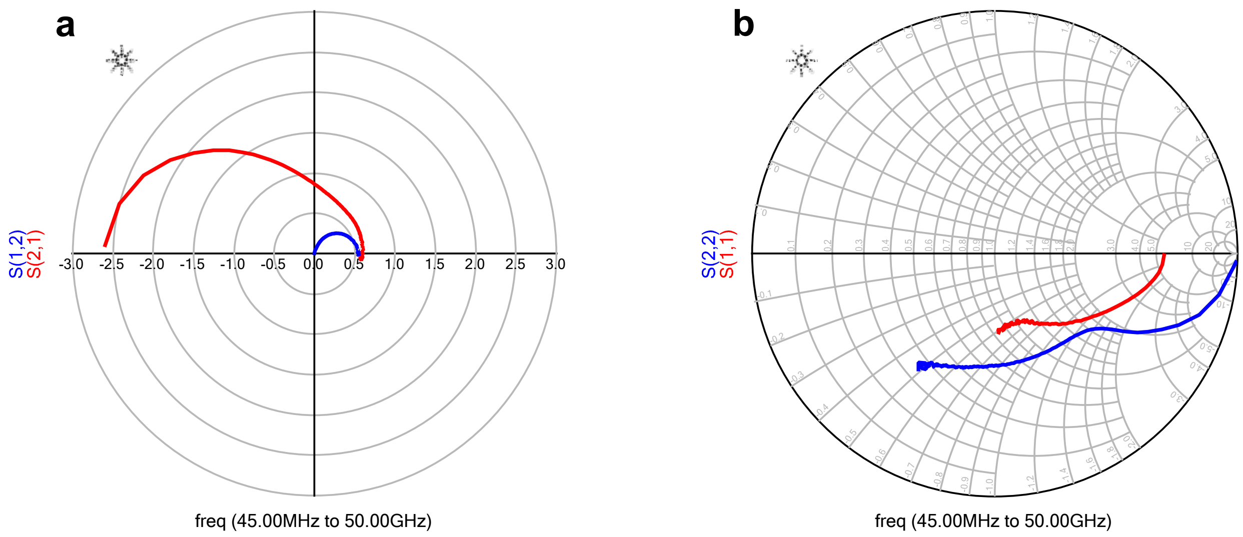

Supplementary Figure 7 | Measured S-parameters of the HBT on a CNF substrate with (a) S_{12} (blue) and S_{21} (red) plotted on a polar plot, and (b) S_{11} (red) and S_{22} (blue) plotted on a Smith chart.

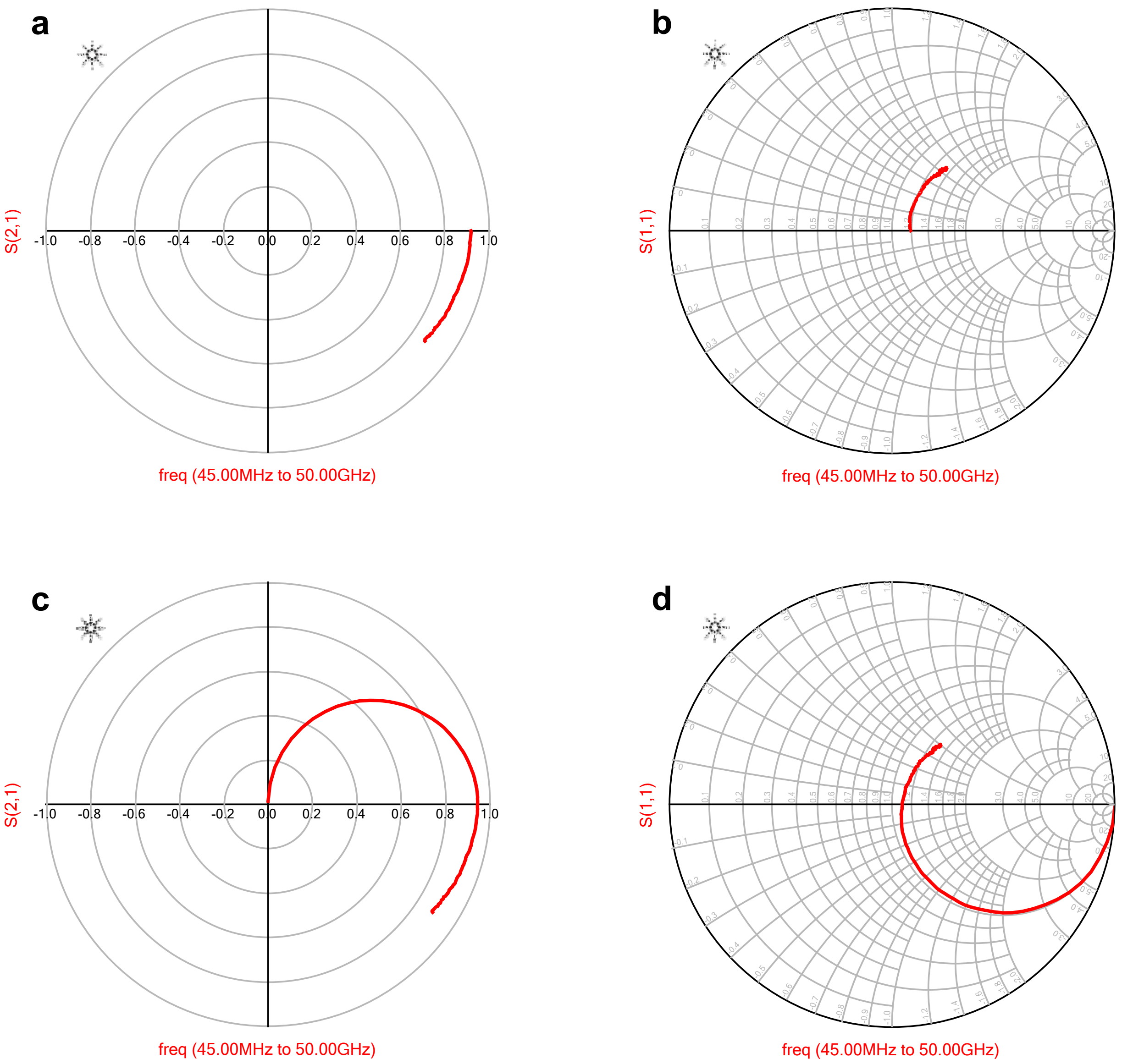

Supplementary Figure 8 | Measured S-parameters of the Schottky diode on a CNF substrate. Figure (a) shows S_{21} plotted on a polar plot while (b) shows S_{11} plotted on a Smith chart under forward bias. Figure (c) shows S_{21} plotted on a polar plot while (d) shows S_{11} plotted on a Smith chart under reverse bias.

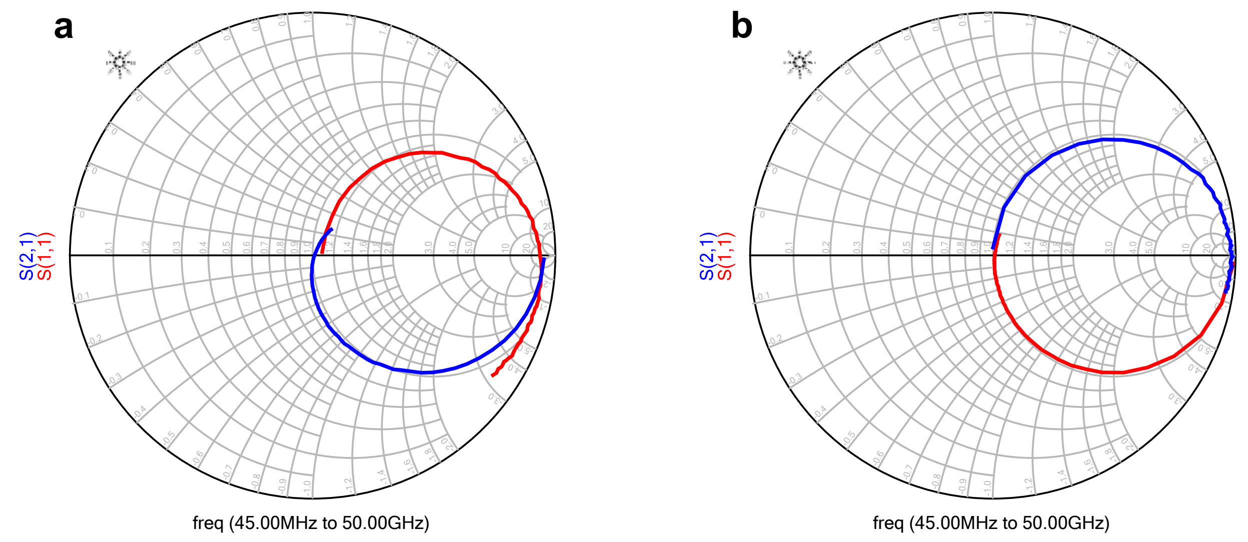

Supplementary Figure 9 | (a) Measured S_{11} (red) and S_{21} (blue) of the inductor on CNF plotted on a Smith chart. (b) Measured S_{11} (red) and S_{21} (blue) of the capacitor on CNF plotted on a Smith chart.

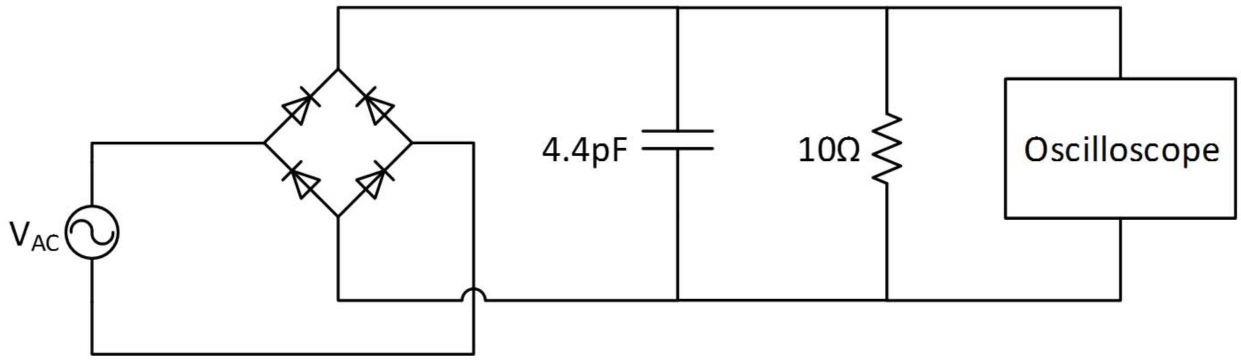

Supplementary Figure 10 | Circuit diagram of the rectifier built on a CNF film.

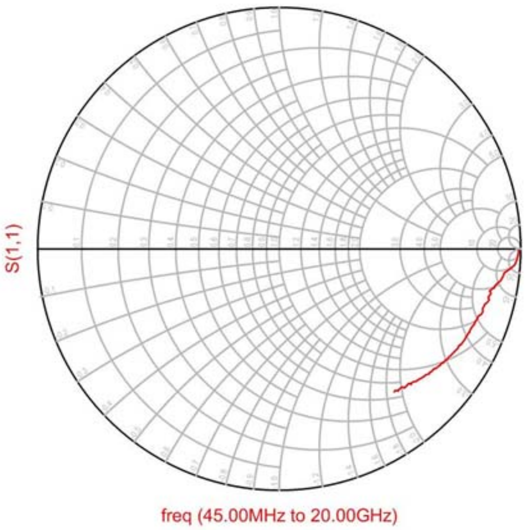

Supplementary Figure 11 | Measured S_{11} of the rectifier at the RF input port plotted on a Smith chart.

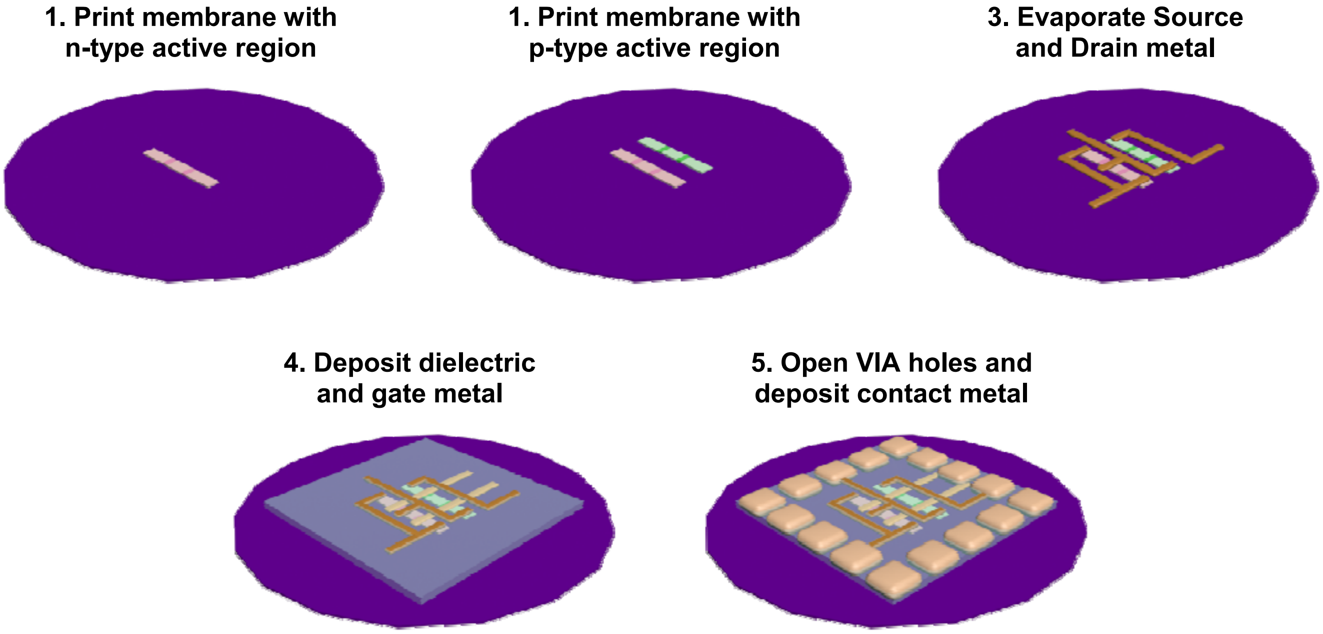

Supplementary Figure 12 | A sequence of schematic illustrations depicting the fabrication process of digital electronics on a temporary Si substrate.

**Target**	**Initial substrate** [Type/Si thickness/Box thickness]	**Implantation condition** [Dopant/Dose/Energy/Tilt angle]	**Screen oxide thickness**	**Anneal condition** [Temperature/Gas/Time]
NMOS Background	p-type/ 205 nm/ 400 nm	Boron (P)/ 4×10^{12} cm^{-1}/20 keV/ 7 °	20 nm	950 °C / N_2, O_2 / 30 min
NMOS Source & Drain	p-type/ 205 nm/ 400 nm	Phosphorus (N++)/ 5×10^{15} cm^{-1}/20 keV/ 7 °	20 nm	950 °C / N_2, O_2 / 10 min
PMOS Background	n-type/ 260 nm/ 600 nm	Phosphorus (N)/ 4×10^{12} cm^{-1}/20 keV/ 7 °	20 nm	950 °C / N_2 / 5 min
PMOS Source & Drain	n-type/ 260 nm/ 600 nm	Boron (P++)/ 1×10^{15} cm^{-1}/10 keV/ 7 °	20 nm	950 °C / N_2 / 5 min

Supplementary Table 1 | Ion implantation conditions for digital electronics.
